# Macroscale patterns in body size of intertidal crustaceans provide insights on climate change effects

**DOI:** 10.1371/journal.pone.0177116

**Published:** 2017-05-08

**Authors:** Eduardo Jaramillo, Jenifer E. Dugan, David M. Hubbard, Heraldo Contreras, Cristian Duarte, Emilio Acuña, David S. Schoeman

**Affiliations:** 1 Instituto de Ciencias de la Tierra, Universidad Austral de Chile, Valdivia, Chile; 2 Marine Science Institute, University of California, Santa Barbara, California, United States of America; 3 Instituto de Fomento Pesquero, Putemún, Chile; 4 Departamento de Ecología y Biodiversidad, Facultad de Ecología y Recursos Naturales, Universidad Andrés Bello, Santiago, Chile; 5 School of Science & Engineering, University of the Sunshine Coast, Sippy Downs, Queensland, Australia; 6 Centre for African Conservation Ecology, Department of Zoology, Nelson Mandela Metropolitan University, Port Elizabeth, South Africa; College of Charleston, UNITED STATES

## Abstract

Predicting responses of coastal ecosystems to altered sea surface temperatures (SST) associated with global climate change, requires knowledge of demographic responses of individual species. Body size is an excellent metric because it scales strongly with growth and fecundity for many ectotherms. These attributes can underpin demographic as well as community and ecosystem level processes, providing valuable insights for responses of vulnerable coastal ecosystems to changing climate. We investigated contemporary macroscale patterns in body size among widely distributed crustaceans that comprise the majority of intertidal abundance and biomass of sandy beach ecosystems of the eastern Pacific coasts of Chile and California, USA. We focused on ecologically important species representing different tidal zones, trophic guilds and developmental modes, including a high-shore macroalga-consuming talitrid amphipod (*Orchestoidea tuberculata*), two mid-shore scavenging cirolanid isopods (*Excirolana braziliensis* and *E*. *hirsuticauda*), and a low-shore suspension-feeding hippid crab (*Emerita analoga*) with an amphitropical distribution. Significant latitudinal patterns in body sizes were observed for all species in Chile (21° - 42°S), with similar but steeper patterns in *Emerita analoga*, in California (32°- 41°N). Sea surface temperature was a strong predictor of body size (-4% to -35% °C^-1^) in all species. Beach characteristics were subsidiary predictors of body size. Alterations in ocean temperatures of even a few degrees associated with global climate change are likely to affect body sizes of important intertidal ectotherms, with consequences for population demography, life history, community structure, trophic interactions, food-webs, and indirect effects such as ecosystem function. The consistency of results for body size and temperature across species with different life histories, feeding modes, ecological roles, and microhabitats inhabiting a single widespread coastal ecosystem, and for one species, across hemispheres in this space-for-time substitution, suggests predictions of ecosystem responses to thermal effects of climate change may potentially be generalised, with important implications for coastal conservation.

## Introduction

Climate change is considered to be one of the most significant contemporary threats to maintenance of global biodiversity, with major consequences predicted for distribution and abundance and ultimately the structure and function of plant and animal communities [1–5]. Effects of climatic warming are already apparent in a wide variety of terrestrial and aquatic taxa in ecosystems ranging from tropical to polar latitudes and across elevational gradients (e.g. [6, 7]). In the ocean, reported effects of increased water temperatures extend from the tropics to the poles [6, 8–14], with consequences for biodiversity, food webs, ecosystem functioning and the provision of ecosystem goods and services. Although the absolute rate of warming may be slower in the ocean, marine ecosystems may change very rapidly in response to climate forcing [15, 16] and coastal marine ecosystems are warming faster than the open ocean (e.g. [17]). For this reason, understanding potential effects of anthropogenic climate change on coastal ecosystems is an increasingly urgent challenge (e.g. [6]). Predicting ecological responses to the effects of altered water and air temperatures, sea level rise, coastal squeeze, storminess and acidification on organismal, population and community processes is particularly important for coastal ecosystems which occupy a narrow strip at the edge of land and sea [18–21].

Body size is increasingly recognized as an important biotic response to the impacts of climate change (e.g. [22–23]). Body size scales strongly with key demographic characteristics, including growth rates, fecundity and survival. These attributes carry significant implications for community- and ecosystem-level responses to climate forcing, and therefore, in turn, for population size and viability, important considerations in conservation planning. Evidence that warming temperatures are linked to reduced body sizes of ectotherms is accumulating [22–28] and these effects appear to be stronger in aquatic than in terrestrial animals [29]. There is also evidence for a wide range of responses of body size to latitude and temperature among some marine invertebrate taxa, between hemispheres and among coastlines, suggesting that the mechanisms driving interspecific patterns in body size may vary strongly across regions and taxa [30]. Importantly, many of these analyses of body size responses have been conducted across temporal scales ranging from decades to millennia (e.g. [24, 25, 27]) that necessarily include potential factors other than temperature, which may also affect body size (e.g. competition and predation regimes, food availability, fishing pressure, pollution) (e.g. [31, 32]). In addition, long-term datasets, museum specimens and fossil evidence suitable for these analyses are not available for many species or ecosystems, particularly for coastal areas, necessitating alternative approaches to body size analyses [33].

Broadly distributed intertidal marine species or taxa, whose distributional ranges include a wide span of environmental variability, may be less vulnerable to extinction from the effects of climate change than those with restricted ranges. However, body size, life history and other attributes of these wide-ranging coastal taxa (e.g. [34–37]) may be quite sensitive to climate [29], particularly near the edges of their range. At a population level these attributes are useful for exploring potential responses to climate change that could carry implications for communities, food webs and ecosystem functioning and provide valuable information for use in conservation strategies. Substitution of space-for-time in comparisons of contemporary macroscale patterns in coastal species may also provide useful new insights on population-level responses to temperature change for a wider range of species and ecosystems, particularly those for which sources of long-term data are insufficient or lacking [33, 38, 39]. These types of spatial comparisons along environmental gradients may also potentially be less affected by environmental or anthropogenic drivers that operate over longer time scales.

Sandy beaches are the most widely represented coastal ecosystem along the temperate coasts of the Eastern Pacific and much of the world [40]. Nonetheless, as narrow fringing ecosystems, beaches are at particular risk of significant impacts from global climate change, sea level rise and coastal development, yet studies of potential ecological responses of these highly dynamic but vulnerable coastal ecosystems to climate forcing are remarkably sparse [21, 41]. However the unique and highly mobile intertidal fauna of sandy beaches [19] makes these often overlooked coastal ecosystems ideal for investigating ecological responses to global climate change. In contrast to the sessile or sedentary intertidal organisms of rocky or muddy shores, the intertidal animals of sandy beaches actively move up and down the shore to adjust to tide and wave conditions [42]. This attribute makes possible geographic comparisons that are relatively unaffected by differences in tidal height among sites or by additional potentially confounding factors associated with latitude and other factors [43].

Strongly influenced by highly productive eastern boundary current upwelling systems [44], the extensive Pacific coast beaches of North and South America are ideal for macroscale comparisons of body size. Intertidal communities of sandy beaches along Chile and California have similar trophic structure and are both taxonomically dominated by mobile crustaceans including talitrid amphipods, cirolanid isopods and anomuran crabs, that make up a high proportion of community abundance and biomass in both hemispheres [45, 46]. Many of these characteristic intertidal invertebrate taxa occupy broad latitudinal ranges on beaches along the coasts of Chile and California, creating an excellent opportunity to evaluate hypotheses concerning macroscale patterns in body size with respect to latitude and ocean temperatures, and to gain potential insights on coastal ecosystem responses to climate change.

To investigate macroscale patterns of intraspecific variation in body size of widely distributed species in relation to latitude and water temperature, we compared populations of ecologically important intertidal crustaceans of sandy beaches along the Chilean and Californian coasts. To expand the generality of our analyses and explore hypotheses concerning the responses of body size for taxa that occupy different tidal zones and differ in feeding and developmental mode, we compared four species that were representative of the broader intertidal community. Species selected for study included a high-shore detritivore, the talitrid amphipod, *Orchestoidea tuberculata* (Nicolet), and two mid-shore scavenging cirolanid isopods, *Excirolana hirsuticauda* Menzies *and E*. *braziliensis* Richardson from Chile, and a swash-zone-dwelling suspension-feeding hippid crab, *Emerita analoga*, from both Chile and California.

## Methods

No specific permits were required for the described field studies. The sandy beaches along the study area are unrestricted to public access and use, and are not privately owned or designated as protected areas (reserves or parks). Similarly, no protected or endangered species were involved in this study.

### Study area

**Fig 1:**
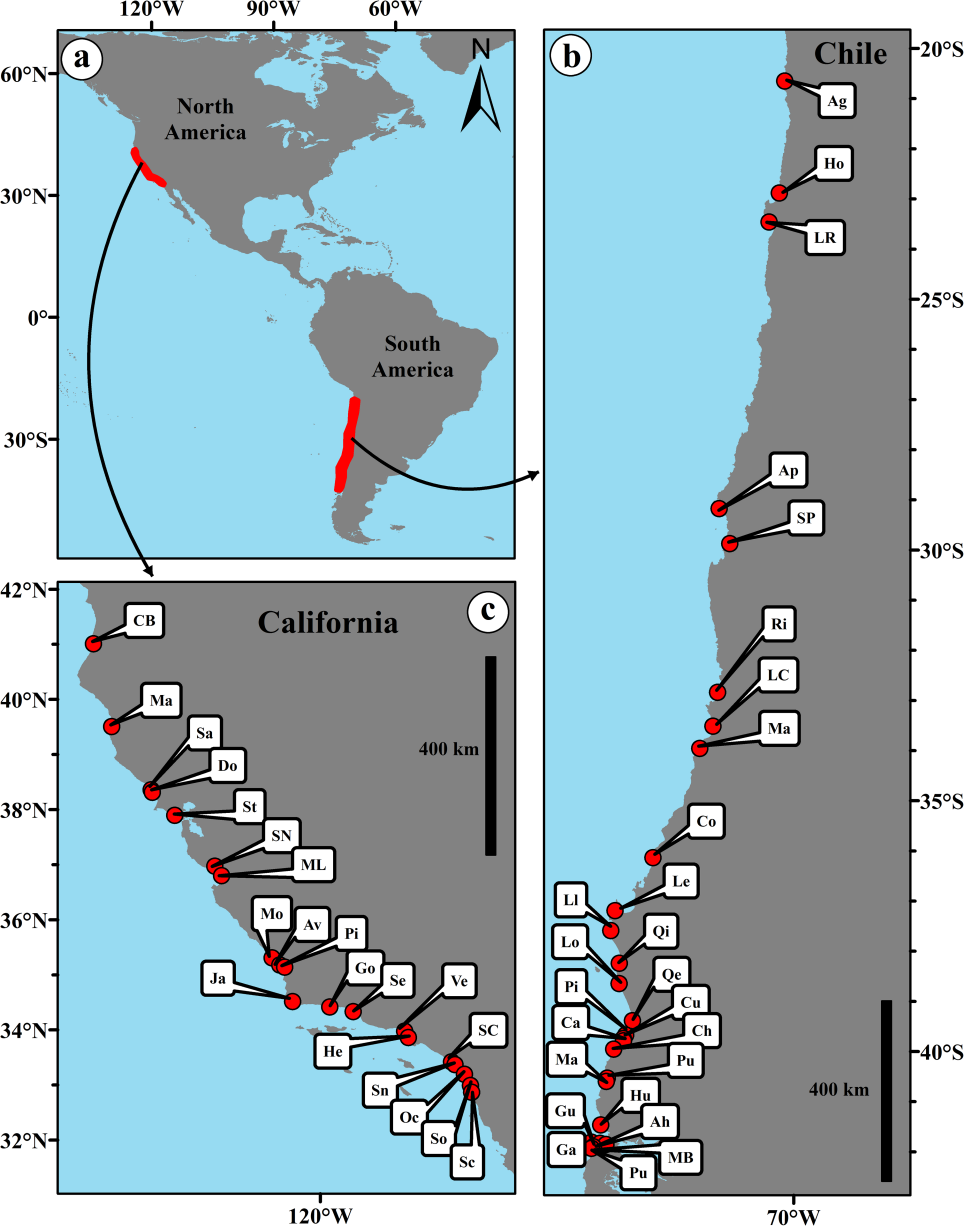
Location of the study beaches along the temperate coasts of the Eastern Pacific Ocean in Chile and California (a). The letter codes for Chile (b) and California (c) correspond to locations in S1 Table.

The 44 sandy beaches we studied extended across 21° of latitude (2300 km) in Chile and 8° of latitude (1000 km) in California (Fig 1A). This represents 16% of the global latitudinal range and 26% of the latitudinal range for eastern Pacific shorelines (Fig 1). For Chile, the northernmost beach was El Aguila (ca. 21°S), while the southernmost beach was Puñihuil (ca. 42°S) (Fig 1B). Most of the Chilean coastline studied is included in the Peruvian or Transition Zoogeographic Zone, which has a southern limit at about 42°S (Isla de Chiloé) [47]. On the coast of California, the northernmost beach sampled was Clam Beach (ca. 40° N) and the southernmost was Scripps Beach (32° N) (Fig 1C), (see [37] for details), spanning three biogeographic faunal provinces for marine organisms, the Oregonian province, the Californian province and the Oregonian/Californian transition zone (e.g. [48]).

### The study organisms

To investigate macroscale variation in body size and life history, we sampled populations of four widely distributed intertidal crustacean species that inhabit different zones of the sandy beach and differ in their feeding and developmental modes. The talitrid amphipod *Orchestoidea tuberculata* is an upper-intertidal semi-terrestrial detritivore that primarily consumes macroalgal wrack [49]. The cirolanid isopods *Excirolana braziliensis* and *E*. *hirsuticauda* are scavengers that feed on animal carcasses stranded on the middle intertidal zones [50]. The hippid crab *Emerita analoga*, is a suspension feeder of the wave swept swash-zone. The three peracarid species are direct-developing brooders with no planktonic larval or adult stages [49, 51] that largely depend on the reproductive output of resident populations. *E*. *analoga* has free-swimming larvae with a lengthy planktonic phase (three to four months) followed by a post larval megalopal stage that settles in the intertidal zone of beaches [52–54]. *O*. *tuberculata* and *E*. *hirsuticauda* are found only along the Chilean coast between 30° and 40°S [55]. *E*. *braziliensis* inhabits both coasts of Central and South America between about 20°N and 41°S and 35°S, on the west and east coast of South America, respectively [56–58]. The geographic distribution of *E*. *analoga* is amphitropical, spanning a region stretching from the Kodiak Islands in Alaska (58°N) to the south coast of Chile (55°S), with an interruption in tropical regions [59].

### Sources and collection of data

We analyzed three sources of data detailed below. Each dataset was collected in the summer over constrained temporal scales to reduce variation due to seasonal patterns in body size of these populations in our comparisons (e.g. [60]):

i) Data from quantitative intertidal samples of *O*. *tuberculata*, *E*. *braziliensis*, *E*. *hirsuticauda* and *E*. *analoga* collected during spring low tides of December 1998—January 1999 at the Chilean beaches of El Aguila, Hornitos, Apolillado, Las Cruces, Matanzas, Cobquecura, Calfuco and Mar Brava (Fig 1B). We focused on these crustaceans, since they are the most common taxa along Chilean sandy beaches and account for most of the abundance and biomass [45, 50, 55]. Sediments were collected with plastic cylinders (25 cm in diameter) at ten equally spaced levels (stations) along four replicated transects (1 m apart) extending from above the drift line to the swash zone; *i*.*e*. the uppermost station of each transect was located above the drift line, the second on that line and the last at the lowest limit of the swash zone indicated by wave-bore collapse. The sediment was sieved through a 1-mm mesh sieve and the specimens collected were stored in 5% formalin until sorted and measured in the laboratory. Body size (length) and sex was determined for all specimens of all species. For *O*. *tuberculata*, body length was defined as the distance from rostrum tip to telson base, whereas for *E*. *braziliensis* and *E*. *hirsuticauda*, body length was defined as the distance from rostrum tip to telson tip. Body size of *E*. *analoga* was defined as carapace length (CL), and was measured with calipers from the tip of the rostrum to the distal scoop of the carapace.

ii) Data from qualitative samples of *E*. *analoga* collected during spring low tides of December 1999—February 2000 at 23 sandy beaches along the Chilean coast (no samples were collected at El Aguila, Hornitos and El Apolillado; Fig 1B). Samples were collected from visible aggregations of *E*. *analoga* in the swash zone using a shovel. All crabs were separated from the sand by sieving through a 1-mm mesh. The sampling continued for at least 20 minutes at each site until more than 100 crabs representing a full range of sizes were collected. The specimens collected were stored in 5% formalin until sorted and measured in the laboratory.

iii) Data from qualitative samples of *E*. *analoga* collected during low tides of July—August 1986 at 20 sandy beaches along the coast of California (Fig 1C) extracted from Dugan et al. [37]. Population samples were collected from visible aggregations in the swash zone with a shovel and by hand as describe above. Crabs were extracted from the sand by sieving through a 1.5 mm mesh and maintained alive for measurement of size and reproductive characteristics within 24 hours. Where present, the morphologically distinct settlement stage or megalopae of *E*. *analoga*, were separated and preserved in 70% ethanol for later measurements in the laboratory.

Sea-surface temperatures (SST) were measured in the surf zone with a mercury thermometer (0.1° C precision) and coincidentally with the samples of crustaceans collected along the Chilean and Californian coasts. Water samples for Chlorophyll *a* analyses were obtained from the surf zone at the Chilean sandy beaches during the period December 1999-February 2000 (see [61] for analytical procedures).

We also measured the physical features of beaches that could potentially influence intertidal biota and/or exhibit latitudinal variability, such as grain size, beach face slope and beach morphodynamic types (see below). Sediment samples for grain-size analysis were collected in Chile with a 3.5-cm diameter plastic core from the uppermost 4 cm of sand of the effluent line and from the lowest swash level. These samples were analyzed with an Emery settling tube. The morphology of each sampling site (*i*.*e*., beach face slope) was determined by Emery´s profiling technique [45]. Wave height was estimated by measuring the height of breaking waves (n = 10) with graduated poles against the horizon, and adding the result to the height difference between the location of the observer and the lowest point where the backwash met the next incoming swash bore. The wave period (measured with a stop watch) was the average time interval between breaking waves. Details for the corresponding methods employed on the Californian coast were similar [37].

### Ancillary SST and Chl a datasets

For SST, we used daily 0.25°-resolution Optimum Interpolation Sea Surface Temperature (OISST) based on Advanced Very High Resolution Radiometer (AVHRR) Satellite data from NOAA’s National Centers for Environmental Information portal (ftp://eclipse.ncdc.noaa.gov/pub/OI-daily-v2/NetCDF/%4i/AVHRR/). We extracted and averaged daily data for pixels having centres within 35 km of the recorded coordinates for each beach site for the periods of 1 Jan 1998 to 31 Dec 2000 (Chile) and 1 Jan 1986 to 31 Dec 1986 (California). For Chlorophyll-a, we used 0.1°-resolution SeaWiFS monthly means from NOAA’s ERDDAP servers (http://coastwatch.pfeg.noaa.gov/erddap/index.html). We extracted and averaged these data for areas spanning 0.5° (latitude and longitude) centered on the coordinates for each beach. Chlorophyll-a data were available only for the Chilean surveys because the California surveys predated the SeaWiFS satellite. For each beach, the mean (Mean), minimum (Min) and Maximum (Max) sea-surface temperature (SST) and chlorophyll-a concentration (CHLa) were tabulated for the years of each of the survey periods (1986 (California SST only), 1998–1999 (Chile), 1999–2000 (Chile).

### Data analyses

#### Beach characteristics

Mean grain size and sorting were calculated according to the moment’s computational method [62]; both are expressed in Phi units (Phi = —log_2_ x grain size in mm; [63]. Mean grain size was used to estimate sand fall velocity (see [64]). An index of sediment diversity was calculated with the Shannon diversity index as used by Dugan and Hubbard [65]. Estimated mean wave height, wave period and sand fall velocity of sediments from the lowest swash level, were used to calculate a dimensionless index of beach morphodynamic state, Dean´s parameter (Ω; [66].

#### Life-history characteristics

Analyses of life-history characteristics of *E*. *analoga* included: body size of the largest male, body size of the smallest and largest ovigerous females, size at maturity of female crabs, and size at settlement (mean size of megalopae; California coast only). The smallest size of ovigerous females corresponded to the 5^th^ percentile size determined from the cumulative number of crabs, while largest size of ovigerous female and male crabs corresponded to the 95^th^ percentile size. The use of percentiles for body sizes allowed us to minimize influence of extreme values and polymodal size distributions characteristic of populations such as those of *E*. *analoga* (cf. [37]). Size at maturity of female crabs was estimated by the smallest size class (1 mm each) at which 50% of the female crabs were ovigerous for each beach sample [36].

#### Predictors of body size

After exploring the possible presence of spatial autocorrelation in our data, we used simple OLS (ordinary least squares) regression analysis to evaluate possible relationships between body sizes of the life stages of the species studied and latitude and surf zone water temperature (SST). For each fitted model, we inspected the residuals visually, using standard diagnostic plots to assess violations of model assumptions. Almost without exception, we found little evidence of severe heteroscedasticity, trends or non-normality among residuals (bearing in mind that sample sizes are small). In the rare instances where such violations were observed, we decided to retain the linear model fit for the sake of consistency. In this small number of cases, our estimated model fit would be slightly worse than it might have been, had we resorted to a transformation of the response, and this only serves to render our results slightly conservative (i.e., we err on the side of Type-II errors, so we do not report spurious relationships as a result of our decision).

Multiple regression analyses (forward stepwise) were used to examine whether composite indices of beach features (sediment index and Dean´s parameter) produced better predictions of body size than surf zone water temperature (SST) for the body size data collected during December 1998- January 1999 and b) life history characteristics of *E*. *analoga*, and environmental variables (summer 1999–2000). The resulting models were compared with the Akaike Information Criterion (AICc) using R [67]. We first fit an intercept-only model as a Null or starting model then allowed the routine to try all possible predictors one at a time, until no more predictors can be added then we picked the model that yielded the lowest AICc. This procedure was repeated for each intertidal crustacean species and each life history characteristic of *E*. *analoga*.

#### Fecundity and biomass estimates

To illustrate the potential consequences of changes in body size on reproductive output and biomass, we used the body size and temperature relationships we obtained to calculate the proportional change in size predicted for mid-sized females at 16°C (the median surf zone SST) as a function of changes in sea-surface temperature ranging from +2°C to -2°C in one degree increments. We then used these proportional changes in body size of a mid-sized female (8.5 mm) to estimate associated changes in 1) fecundity of the direct-developing brooding isopod *E*. *hirsuticauda* using length/clutch size relationships [51] and 2) fecundity and biomass of the suspension-feeding primary consumer *E*. *analoga* (21.4 and 24.0 mm CL for Chile and California, respectively) using length/biomass and length/clutch size relationships for this species in Chile and California [36, 68].

## Results

### Sea-surface temperature and Chlorophyll a

**Fig 2:**
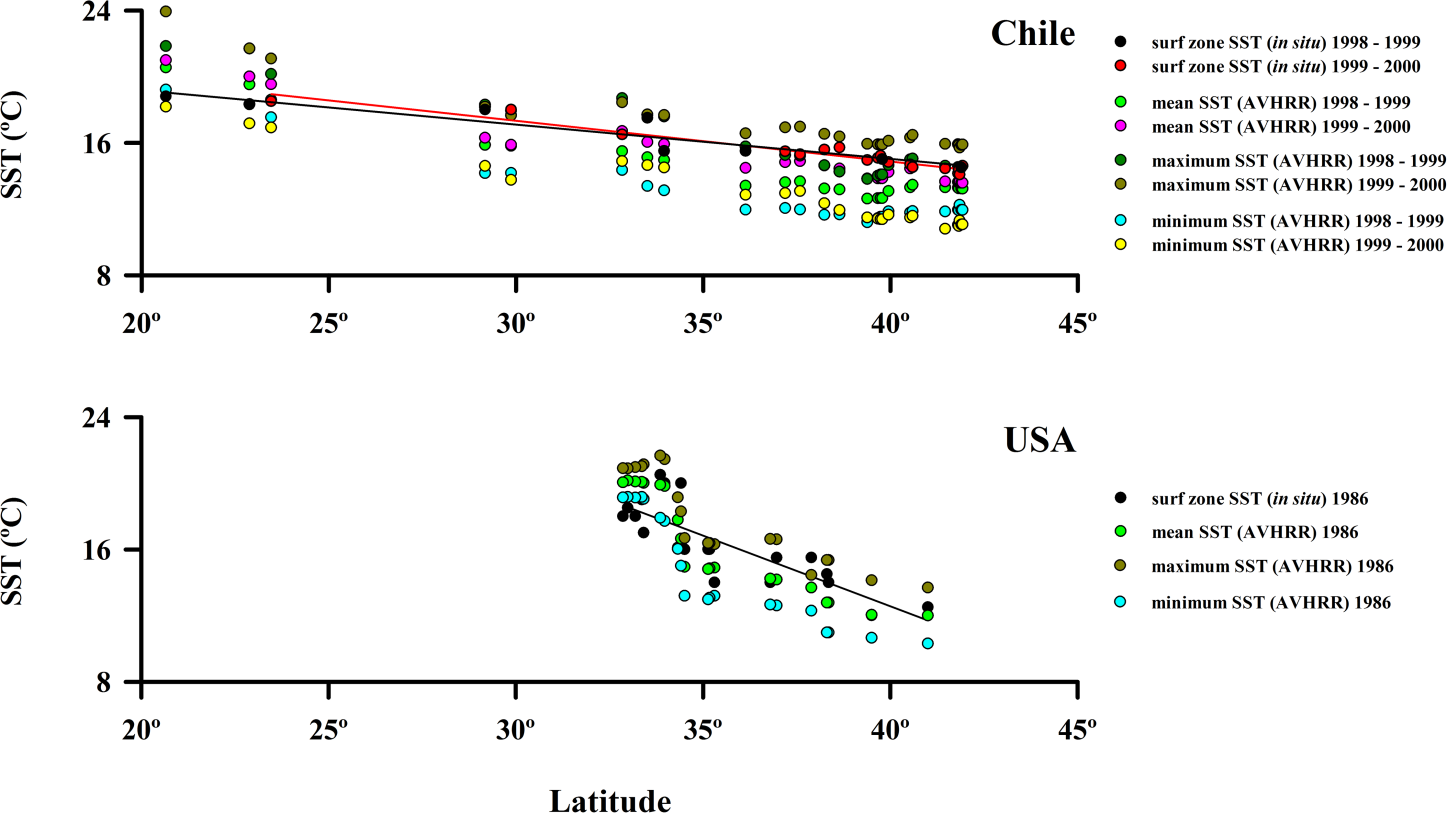
Spatial variability of surf zone SST (*in situ*) and mean, maximum and minimum SST AVHRR along the coast of Chile and USA (California). The regressions for data collected in situ are included, other regressions are in S2 Table.

Sea-surface temperature (SST) measured at the surf zone at the time of population sampling (summer) and values derived from satellite imagery decreased significantly with increasing latitude in the study regions in both hemispheres (Fig 2 and S2 Table): 1) for Chile, the observed range of surf zone water temperature was 18.5°C to 14.1°C (SST = 24.711 –(0.246 x latitude), r = 0.95, *P* < 0.001, df = 22); and 2) for California, the range of surf zone water temperatures was greater over a shorter latitudinal gradient, ranging from 12.0°C to 20.5°C and the slope of the relationship with latitude was steeper (SST = 46.751 –(0.855 x latitude), r = 0.82, *P* < 0.001, df = 19). Our field measured SST values were strongly correlated with satellite-derived values for mean, minimum and maximum SST for the respective survey years for Chile and for California (Fig 2 and S2 Table). Overall, sea-surface temperatures at beaches sampled in our study spanned about a third of the range of temperatures present along the eastern shores of the Pacific Ocean (9.5° C of approximately 30° C) [69].

**Fig 3:**
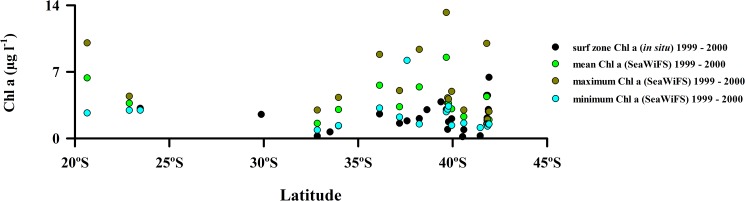
Spatial variability of surf zone Chlorophyll a (*in situ*) and mean, maximum and minimum Chl a SeaWiFS along the coast of Chile and USA (California).

Field-measured surf zone values or satellite-derived values of Chlorophyll a concentrations along the Chilean coast did not show any significant trend with latitude for the survey years (Fig 3 and S2 Table).

### Beach characteristics

**Fig 4:**
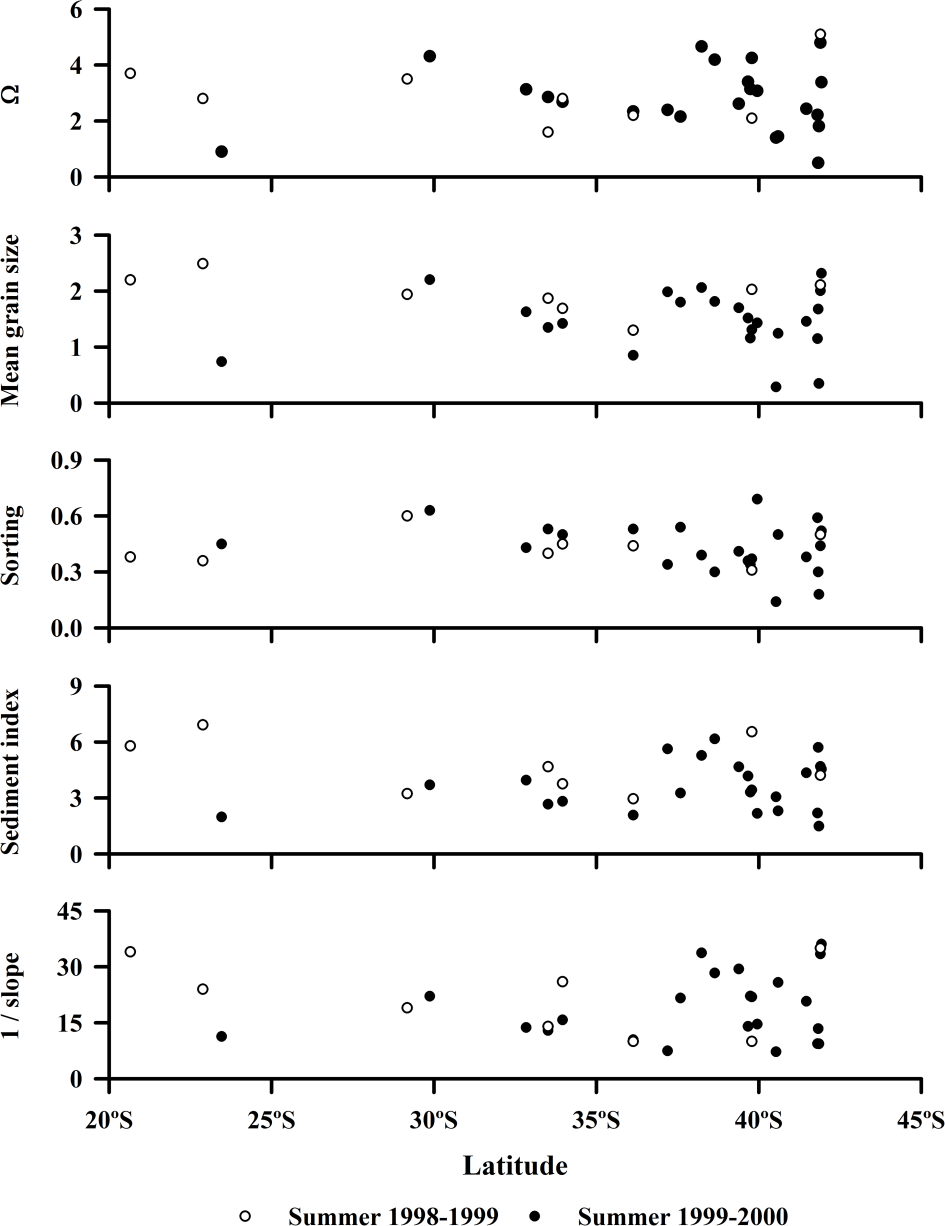
Spatial variability of Dean´s parameter (Ω), mean grain size in Phi units, sorting in Phi units, sediment index, and beach face slope of sandy beaches along the Chilean coast.

No significant patterns in beach characteristics with latitude or coastline distance were evident in our study regions in the southern or the northern hemisphere (S3 Table). Thus, small-scale or local variability among beaches was usually greater than macro-scale or geographic variability in both hemispheres (Fig 4) [37]. In Chile, these analyses included morphodynamic types as defined by Dean´s parameter (Ω), mean grain size and sorting of sands, the sediment diversity index and beach-face slopes (*P* > 0.05) (Fig 2). In California, the analyses included grain size, sorting and sediment index, which were mutually collinear (*P* < 0.05) (see [37]). For Chile, values of Dean´s parameter (Ω) indicates that two of the study beaches were reflective (Ω < 1), while all the others were intermediate in morphodynamic type (Fig 4) (sensu [66]). Intertidal sands were composed primarily of medium-sized grains (Fig 4) (mean grain sizes ranging from 1 to 2 Phi [63]. Values for sediment sorting indicated that most Chilean beaches had well-sorted sands (Fig 4) (< 0.5 Phi of standard deviation) [63]. The sediment diversity index varied between 1.98 and 6.17 (Fig 4), while beach-face slopes varied between 1/8 and 1/36 among beaches (Fig 4). Flatter beaches had finer sands and *vice versa* (beach face slope = 3.336 + (10.711 x Phi), r = 0.65, *P* < 0.001, d.f. = 22).

### Body sizes of populations of intertidal crustaceans in Chile

**Fig 5:**
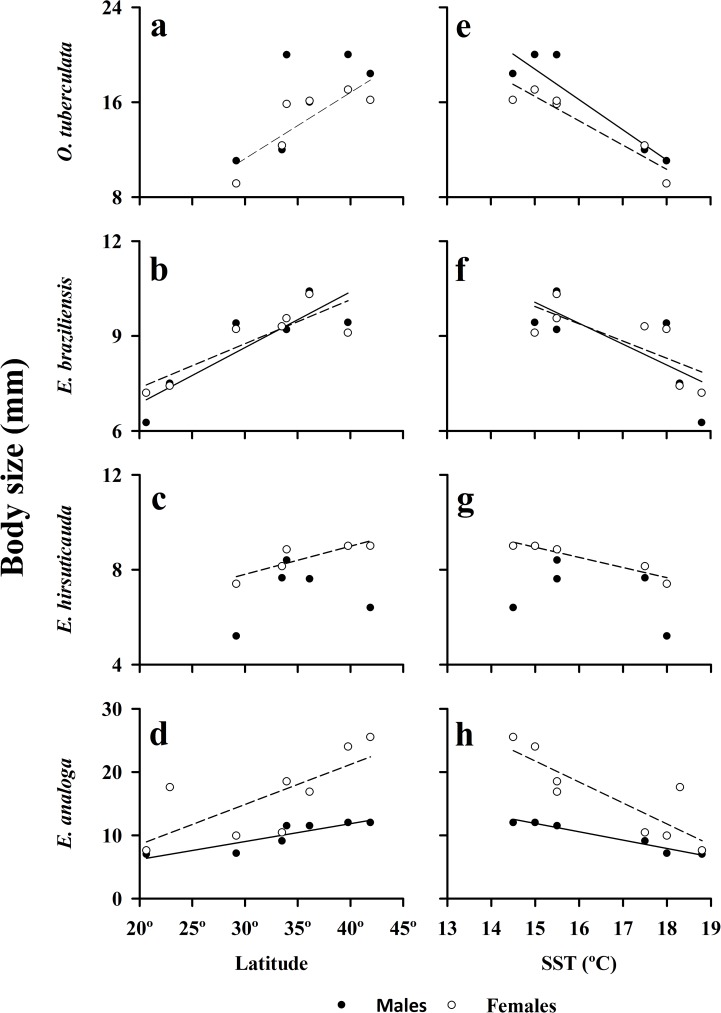
Body sizes of largest males and females of *Orchestoidea tuberculata*, *Excirolana braziliensis*, *Excirolana hirsuticauda* and *Emerita analoga* as a function of latitude and sea-surface temperature (SST) along the Chilean coast (data collected during December 1998—January 1999).

Largest body size (95^th^ percentile) of individuals increased significantly (P <0.05) with latitude for both males and females of the cirolanid isopod *E*. *braziliensis* (Fig 5B), for the hippid crab *E*. *analoga* (Fig 5D), as well as for females of the talitrid amphipod *O*. *tuberculata* (Fig 5A). Latitudinal trends were also evident in the body size of males of *O*. *tuberculata* (Fig 5A) and males of *E*. *hirsuticauda*, but relationships were not statistically significant (Fig 5C) (Table 1). The trend found in the body size of largest females of *E*. *hirsuticauda* was close to significant as well (Fig 5C) (Table 1). The slopes of the relationships of body size and latitude were steepest (>0.5) for the largest male and female talitrid amphipods and the largest female sand crab and much lower (<0.2) for the two scavenging isopods (Table 1).

**Table 1:** Results of regression analyses of body size of intertidal crustaceans as a function of latitude and sea-surface temperature for populations in the southern hemisphere (Chilean coast: data for December 1998—January 1999).

	Intercept	Slope	r	adjusted r	std. error	*P*
			**LATITUDE**			
*O*. *tuberculata*, largest male	-5.85	0.618	0.72	0.40	3.07	0.107
largest female	-5.477	0.557	0.84	0.62	1.89	0.038
*E*. *braziliensis*, largest male	3.347	0.176	0.88	0.72	0.74	0.01
largest female	4.58	0.139	0.85	0.68	0.65	0.014
*E*. *hirsuticauda*, largest male	5.172	0.054	0.20	0.00	1.43	0.749
largest female	4.26	0.118	0.87	0.67	0.40	0.056
*E*. *analoga*, largest male	0.554	0.028	0.89	0.74	1.14	0.008
largest female	-4.101	0.633	0.73	0.46	4.85	0.04
			**SEA-SURFACE TEMPERATURE**			
*O*. *tuberculata*, largest male	57.077	-2.552	0.91	0.79	1.79	0.011
largest female	47.224	-2.047	0.95	0.87	1.12	0.004
*E*. *braziliensis*, largest male	19.978	-0.661	0.73	0.46	1.05	0.061
largest female	18.112	-0.545	0.75	0.47	0.83	0.054
*E*. *hirsuticauda*, largest male	12.173	-0.316	0.37	0.00	1.35	0.540
largest female	15.363	-0.428	0.95	0.88	0.25	0.012
*E*. *analoga*, largest male	31.682	-1.320	0.98	0.95	0.52	<0.001
largest female	71.408	-3.312	0.85	0.68	3.73	0.007

**Fig 6:**
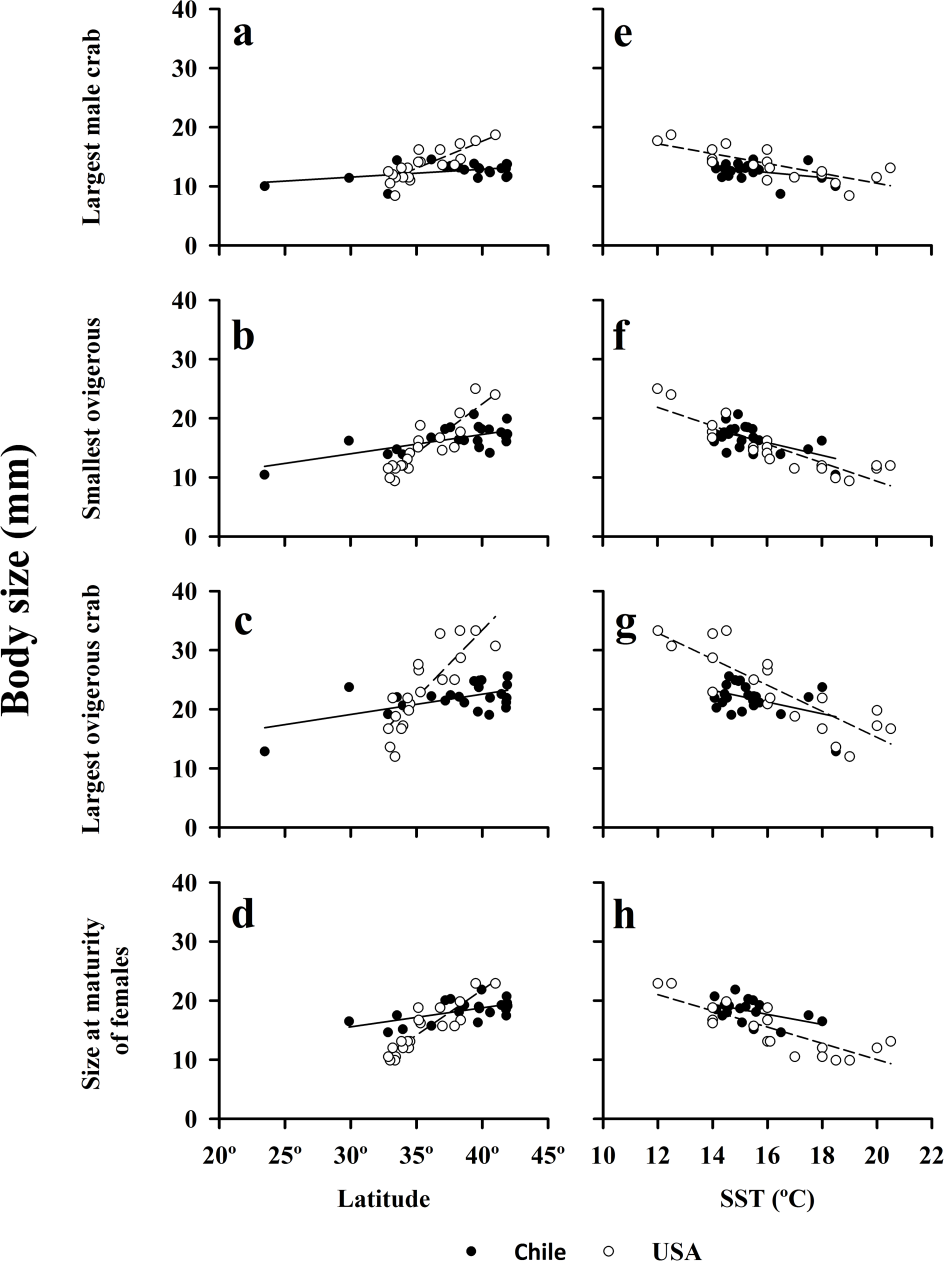
Body size of largest male crabs, smallest and largest ovigerous crabs and size at maturity of females of *Emerita analoga*, as a function of latitude and surf zone temperature (SST) along the Chilean and Californian coasts (data collected during December 1999—February 2000 and July 1986, respectively).

The body sizes of the largest males and females of the amphipod *O*. *tuberculata*, and the crab *E*. *analoga*, were inversely and significantly correlated with surf zone water temperature (SST) (Table 1, Fig 5E and 5H respectively). Life-history characteristics of *E*. *analoga* were significantly correlated with surf zone SST (Table 2, Fig 6E–6H). A similar pattern was also evident for females of the isopod *E*. *hirsuticauda*, but the trend seen in largest males of this isopod was not significant (Fig 5G) (Table 1). A near-significant relationship with surf zone SST was present for the body sizes of the largest males and females of *E*. *braziliensis* (Fig 5F) (Table 1).

**Table 2:** Results of regression analyses for life history characteristics as a function of latitude and sea-surface temperature for populations of *E*. *analoga* in the southern and northern hemispheres (Chilean coast: data from December 1999—February 2000; Californian coast: data from 1986).

Life History Characteristics	Region	Intercept	Slope	r	adjusted r	std. error	*P*
		**LATITUDE**					
Largest male crab	Chile	7.545	0.134	0.45	0.16	1.26	0.04
	California	-19.030	0.916	0.83	0.67	1.51	<0.001
Smallest ovigerous crab	Chile	4.173	0.327	0.67	0.42	0.70	<0.001
	California	-43.846	1.656	0.9	0.80	0.99	<0.001
Largest ovigerous crab	Chile	8.282	0.343	0.58	0.31	2.25	0.004
	California	-57.986	2.284	0.84	0.69	3.60	<0.001
Size at maturity of females	Chile	5.91	0.322	0.6	0.32	1.57	0.006
	California	-39.532	1.534	0.9	0.80	1.84	<0.001
Size at settlement	California	0.780	0.083	0.94	0.88	0.05	<0.001
			**SEA-SURFACE TEMPERATURE**				
Largest male crab	Chile	19.164	-0.426	0.37	0.10	1.31	0.087
	California	27.183	-0.833	0.79	0.60	1.67	<0.001
Smallest ovigerous crab	Chile	33.275	-1,085	0.58	0.30	1.86	0.004
	California	40.601	-1.562	0.88	0.76	2.14	<0.001
Largest ovigerous crab	Chile	37.114	-0.993	0.44	0.15	2.49	0.037
	California	59.377	-2.208	0.84	0.70	3.56	<0.001
Size at maturity of females	Chile	31.32	-0.853	0.47	0.17	1.73	0.039
	California	37.521	-1.376	0.84	0.69	2.29	<0.001
Size at settlement	California	4.615	-0.056	0.73	0.50	0.11	0.002

### Body sizes and beach characteristics in Chile

We found some evidence of relationships between the body sizes of crustacean populations and beach characteristics, particularly for the mid shore isopods. The body sizes of males of *E*. *braziliensis* were correlated with beach face slopes (body size = 11.389 - (0.133 x slope), r = 0.84, *P* = 0.016, df = 22), females of *E*. *braziliensis* with sediment size (body size = 14.161 - (2.738 x sediment size), r = 0.91, *P* = 0.005, df = 22) and sediment index (body size = 11.471 - (0.537 x sediment index), r = 0.76, *P* = 0.050, df = 22) and males of *E*. *hirsuticauda* with sediment sorting (body size = 14.034 - (14.606 x sorting), r = 0.89, *P* = 0.042, df = 22). In general, relationships between body sizes of crustacean populations and beach morphodynamic state (Dean’s parameter) were not significant for data collected in Chile. However, we found a significant relationship between the size of the largest ovigerous females of *E*. *analoga* and Dean’s parameter (body size = 18.557 + (1.177 x Dean’s), r = 0.51, *P* = 0.014, df = 22).

The results of multiple regression analyses (forward stepwise) with Akaike Information Criterion (AICc), showed that in most cases, the inclusion of SST, resulted in a better model to predict body sizes than the null model or those obtained by using only beach characteristics (Table 3). The analyses run with the December 1998—January 1999 data, showed that surf zone SST was the most important predictor of body size in almost all cases; surf zone SST resulted in a better model to predict body sizes of the largest males and females of *O*. *tuberculata* and *E*. *analoga* (also mean grain size for largest females of *E*. *analoga*) (Table 3). Body sizes of largest males of *E*. *braziliensis* were better predicted by variability in beach face slopes while females were better predicted by mean size of sand grain size; while no single parameter studied predicted body size of *E*. *hirsuticauda*, either largest males and females better than the starting model (Table 3). The analyses run with the data for collected for *E*. *analoga* during December 1999—February 2000, showed that surf zone SST was again the most important predictor for body size of this species (Table 3). Other parameters that were also included in the models included: mean grain size for the smallest ovigerous crabs and beach face slope for the largest ovigerous crabs (Table 3).

**Table 3:** Values of multiple regression analyses of body size of intertidal crustaceans (summer 1998–1999), and b) life history characteristics of *E*. *analoga*, and environmental variables (summer 1999–2000) in the southern hemisphere (Chile) (see Methods for details).

response	AICcNull	AICcPred	predictors	DF	F	p
a) December 1998—January 1999						
*O*. *tuberculata*, largest male	40,41749	39,59881	surf zone SST	1, 40	20,274	0,0108
largest female	37,38065	33,98107	surf zone SST	1, 40	33,321	0,004472
*E*. *braziliensis*, largest male	30,57527	28,66613	beach face slope	1, 50	12,853	0,01579
largest female	27,64046	22,66055	mean grain size	1, 50	22,684	0,005047
*E*. *hirsuticauda*, largest male	25,37744		none			
largest female	19,49282		none			
*E*. *analoga*, largest male	37,16391	22,34942	surf zone SST	1, 50	107,82	0,0001427
largest female	58,13819	53,46253	surf zone SST	1, 50	49,984	0,0008757
b) December 1999—February 2000						
*E*. *analoga*, largest male crab	80,25814	79,65559	surf zone SST	1, 20	3,2412	0,08691*
smallest ovigerous crab	105,76383	99,05811	surf zone SST	1, 20	12,0834	0,002383
		97,79387	mean grain size	1, 20	4,0312	0,058367*
largest ovigerous crab	114,52925	109,29312	beach face slope	1, 20	10,2138	0,004539
		107,19009	surf zone SST	1, 20	4,9238	0,03822
size at maturity of females	86,23974	84,15859	surf zone SST	1, 18	4,9688	0,03879

* non significant

### Body sizes of an amphitropical species

**Fig 7:**
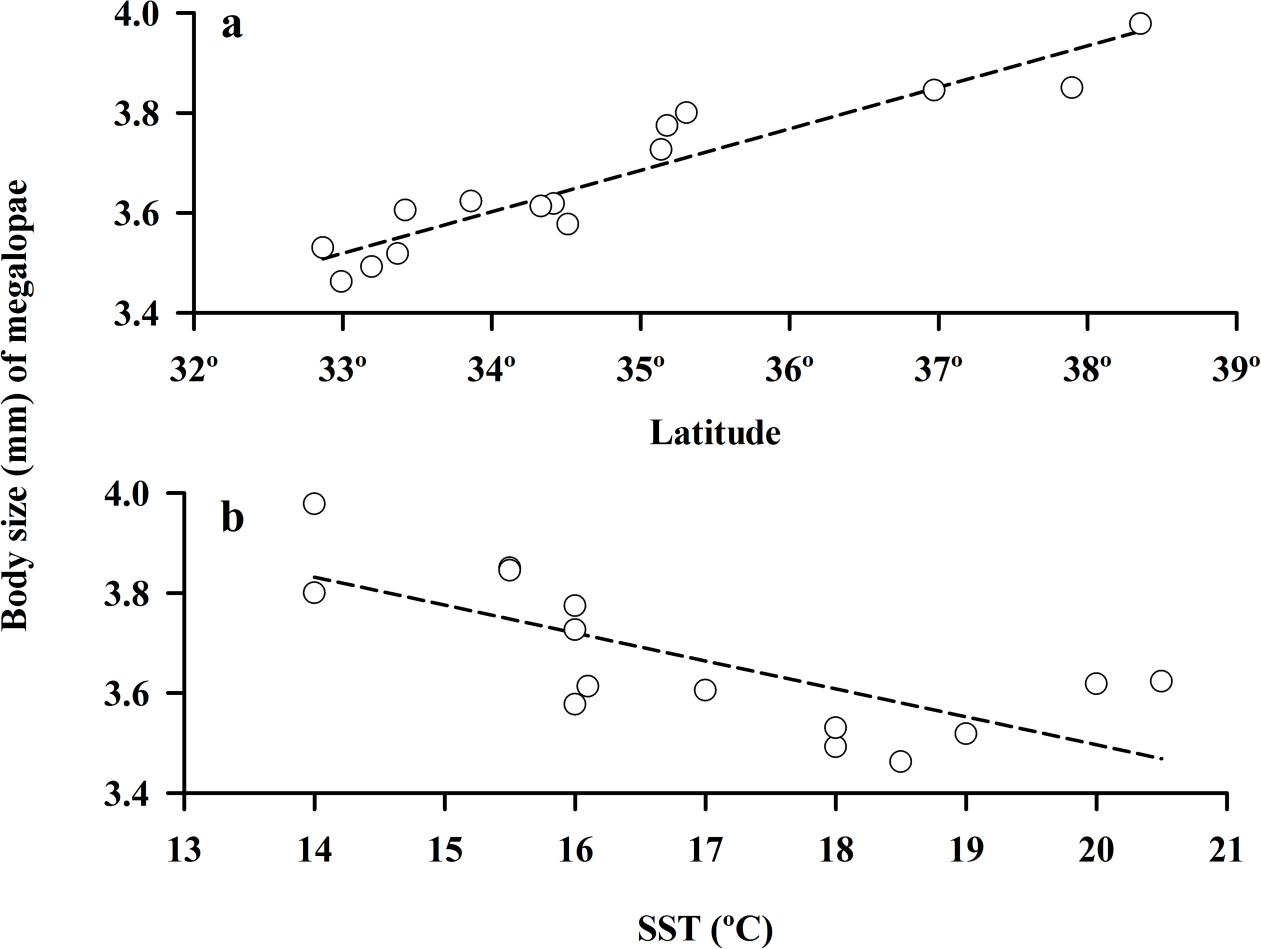
Body size of megalopae as a function of latitude and surf zone temperature (SST) along the Californian coast (data collected during July 1986).

Our comparisons of population characteristics of the amphitropical hippid crab, *E*. *analoga*, in Chile and California, revealed very similar overall macroscale patterns in body size of these populations. The body size of the largest males, smallest and largest ovigerous females, the size at maturity of females, and the mean size at settlement (megalopae) increased significantly (*P* < 0.05) from lower to higher latitudes in both the southern and northern hemisphere (Figs 6A–6D; 7A) (Table 2). In all comparisons, the slope of the observed relationship with latitude was steeper for populations of this crab in the northern hemisphere (>0.9) than the southern hemisphere (<0.35) (Fig 6A–6D, Table 2).

Inverse relationships between the body sizes of all of the adult life stages of *E*. *analoga* populations and sea-surface temperature were evident in both hemispheres, (Fig 6E–6H) (Table 2). The size at settlement (mean size of megalopae) was also inversely correlated with sea-surface temperatures (California only) (Fig 7B) (Table 2). With the exception of the size of the largest male crabs from Chile, these relationships were significant (*P*< 0.05) (Table 2). In all comparisons, the slopes of relationships between body size and water temperature in *E*. *analoga* were more similar between the northern and southern hemispheres (>0.85) (Fig 6E–6H, Table 2) than observed for latitude (Fig 6A–6D) but were always steeper in California.

### Fecundity and biomass estimates

**Fig 8:**
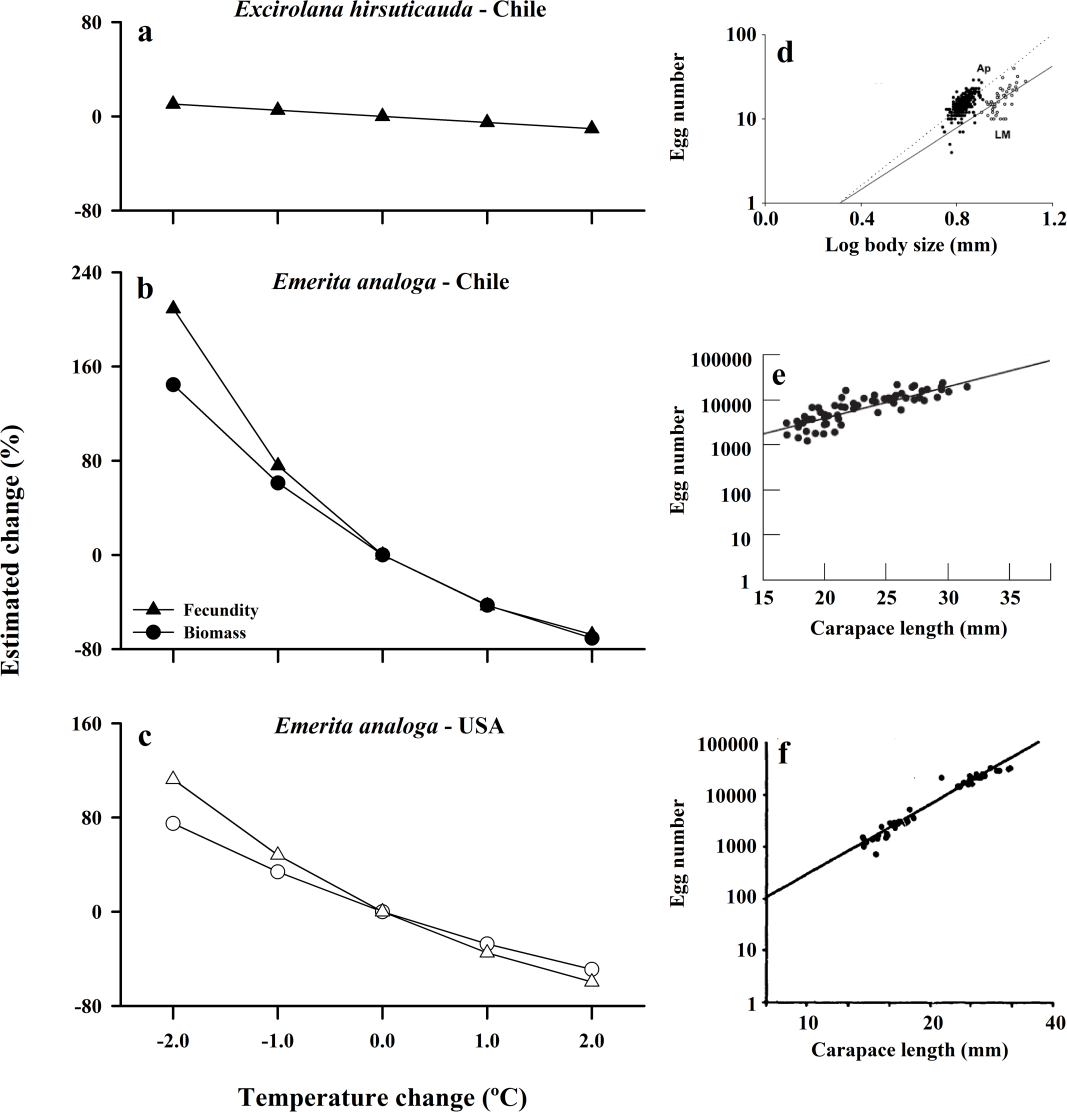
Estimated effect of changing temperature on fecundity (triangles) and biomass (circles) calculated for: a) the isopod *Excirolana hirsuticauda* from Chile, b) *Emerita analoga* from Chile (black symbols), and c) *Emerita analoga* from USA (California) (white symbols). For each species and region, scatter plots and regressions of egg number as a function of body size are shown in the corresponding panels on the right side of the figure. d) fecundity for *E*. *hirsuticauda* was estimated using data collected from La Misión beach (LM), Chile (log fecundity = (0.074 x body size) + 2.07)[68]; e) fecundity for *E*. *analoga* in Chile was estimated using regression of data collected from Mehuin beach, Chile (log fecundity = -0.57 + 1.83 log (carapace length)) [51]; f) fecundity for *E*. *analoga* in California was estimated using regression of data collected at Avila Beach, USA (log fecundity = (4.47 x carapace length)– 2.02) [36]; the biomass equation used in calculations for *E*. *analoga* is from Chile (Biomass = 0.00004 x (carapace length ^ 3.3189)).

Results of our analysis of the potential consequences of changes in body size on reproductive output varied between the crab and isopod, with stronger effects for the larger-sized crab. Through the effects of sea-surface temperature on body size, an increase of 2°C was estimated to result in a moderate ~10% decline in fecundity of *E*. *hirsuticauda* (Fig 8A, S4 Table). For the suspension feeding amphitropical crab, *E*. *analoga*, predicted responses to temperature were nearly 2–3 times larger than in the scavenging isopod, and were comparable across hemispheres (Fig 8B and 8C, S4 Table). An increase in SST of 2°C could thus result in a reduction of body mass of ~71% for crabs from Chile and ~49% for Californian crabs, with corresponding decreases in fecundity of ~68% and ~60%, respectively (S4 Table). Conversely, if sea-surface temperatures cooled by 2°C, as is predicted for some coastal upwelling areas (see [44,70]), body mass of *E*. *analoga* would be expected to increase by ~144% and ~75% for Chile and California, respectively, while individual fecundity would increase by ~200% and ~110%, respectively (Fig 8B and 8C, S4 Table).

## Discussion

Our analyses demonstrate consistent macro-scale patterns of body size for broadly distributed intertidal animals of sandy beach ecosystems in the northern and southern hemispheres of the eastern Pacific coast. Body size increases towards higher latitudes and decreases as sea-surface temperature increases for ecologically important species spanning different trophic groups and life histories. Although our results, and those of Dugan et al. [37], suggest that local habitat features that do not vary with latitude (e.g. beach morphodynamic type) may also contribute to variation in body size in some life stages, most of the variation observed in body size of these intertidal crustaceans was best explained by surf zone water temperature alone.

The relatively strong influence of sea-surface temperature on body size is supported by the similarity of results on adult body sizes and life history characteristics for the amphitropical hippid crab, *E*. *analoga* across hemispheres. The stronger latitudinal response in body size found along the California coast also provides an example of the role of environmental steepness, such as thermal gradients, in shaping broad scale macroecological patterns (e.g. [71]).

The similar responses we observed in the size at settlement (megalopae) and adult body sizes of the hippid crab, *E*. *analoga*. also suggests a strong role of sea surface temperature in these macroscale patterns. At settlement the megalopal post larvae transition from the plankton to the intertidal beach, then spend up to one month in the intertidal at the same size before molting to become juvenile crabs [53, 54]. This means that the body size of this morphologically distinct settlement stage, likely reflects responses to sea-surface temperature rather than to other beach characteristics. The size of settlement of *E*. *analoga* also has important implications for juvenile growth rates due to the strong relationship observed between post-settlement molt increments and body size [72].

Inter- and intraspecific variation in body size with temperature (and latitude) that is generally consistent with patterns predicted by ecogeographical or biogeographical rules [73], such as Bergmann’s Rule [74], James’s Rule [75], the Temperature-Size Rule [76], and others, has been reported for a diversity of animal groups. These include vertebrates (e.g. [77–80]), terrestrial invertebrates (e.g. [77,81]), and marine invertebrates such as mollusks and crustaceans [34, 35, 82–84] as well as two of the species studied here *E*. *analoga* [36, 37, 85] and *E*. *braziliensis* [86]. However, few studies of marine invertebrates have examined macro-scale variability in body size of multiple species from the same coastal ecosystem that possess different feeding habits, microhabitats and life histories, as we have done here. Our finding that body sizes of a suite of ecologically important intertidal crustaceans are strongly correlated with sea-surface temperatures across latitudinal gradients, and for one species, across hemispheres, suggest that more general predictions of ecological responses to climatic variation for coastal ecosystems may be possible, with significant implications for conservation of these threatened ecosystems. In addition, the general lack of evidence supporting a strong role of biotic interactions, such as predation or competition, in structuring intertidal communities of sandy beach ecosystems reported to date [21], makes our results linking body size and life history patterns with environmental forcing more compelling.

Importantly, our results, which extend across co-occurring crustacean species that utilize distinctly different food resources and experience very different levels of submergence by seawater, imply that mechanisms affecting body size can operate in a similar or complementary manner across different functional groups and microhabitats. The mechanisms that drive observed macroscale patterns in body size, and the responses of any particular species, taxon, or group to environmental variation, can be complex and highly context dependent [24, 30, 81, 87]. A variety of environmental mechanisms have been proposed to explain observed patterns of larger body sizes of marine invertebrates towards colder waters. These include spatial variability in water temperature (e.g. [83]), differential growth rates due to variation in productivity [37] and concentration/partial pressure of oxygen in the water [88]. Biotic processes that can co-vary with latitude, such as predation, have also been shown to influence body size patterns in marine invertebrates but the majority of studies, including ours, do not address this potential mechanism separately [87].

For ectotherms such as intertidal invertebrates, increased temperature can be associated with greater metabolic costs for maintenance, leaving less energy available for both growth and reproduction (e.g. [27, 89]). In crustaceans, warm water temperatures can accelerate ovarian maturation, causing ovarian and somatic growth to become antagonistic (see [90]), resulting in smaller sizes at maturity and maximum body sizes [91], more recently termed the Temperature-Size Rule [29, 76]. Food availability and temperature can also have interactive effects on growth and maturation in ecottherms. Although trends of higher productivity with increasing latitude have been described for both the Chilean and Californian coasts (e.g. [92]), these patterns were not detected in our field or satellite-derived chlorophyll *a* data for the study period in Chile. However although we did not find significant relationships between body size of *E*. *analoga* and food availability along the Chilean coast, significant relationships between body size of *E*. *analoga* and food availability were found in a California study [37], suggesting the potential for interactions between food and temperature to influence body sizes.

Our results on macro-scale patterns in body size and life history of intertidal crustaceans from two hemispheres and time periods illustrate how the use of contemporary space-for-time substitutions [33] within a coastal ecosystem can provide new insights on potential population-level responses to temperature shifts associated with global climate change. Integration across the responses of individual species in a community or ecosystem can provide a greater understanding of potential ecological effects of climate change that extends beyond a population or metapopulation level [25,26] and offers valuable insights for ecosystem level responses. For example, while much effort has focused on predicting shifts in species ranges in response to climate change (e.g. [5, 93–95]), far fewer studies have considered the ramifications of altered ecological processes that impinge directly on the planning and implementation of conservation actions [96, 97]. Our results showing similar responses in the body size of several important intertidal species to temperature for the widespread coastal ecosystem of sandy beaches illustrate the importance of such considerations not only for ecology, but also for conservation and management.

Importantly, our comparisons suggest that changes in ocean temperatures of even a few degrees °C associated with global warming or other climatic variability are likely to have significant effects on body size (~4% - 35% length °C^-1^) in marine intertidal populations. Further, the variation in body size associated with an increase of 2°C in sea-surface temperatures resulted in proportional effects on individual biomass (49–71%) and fecundity (10% - 68%) of these species with stronger effects on the larger crab species. Even assuming population densities are constant, such effects of altered body size have consequences for population biomass, reproductive output and demography that carry implications for the structure and function of the affected communities and ecosystems. For example, the crab, *E*. *analoga*, often comprises the majority (>50%) of the intertidal biomass on sandy beaches and represents an important prey resource for birds and fish [46]. Thus, reductions in body size and altered population characteristics of this species predicted in response to increased temperature would also significantly reduce overall intertidal community biomass and affect the ecosystem function of food-web support for coastal birds and fish, including species of conservation significance. Talitrid amphipods, such as *O*. *tuberculata*, are important intertidal consumers of stranded macroalgal wrack, playing a key role in the breakdown and remineralization of this subsidy from coastal reefs and kelp forests worldwide [98]. Changes in the size structure of these populations can strongly affect rates of consumption and processing of macroalgae and the ecosystem function of beaches in coastal nutrient cycling [98].

In summary, alterations in ocean temperatures associated with global climate change are expected to strongly affect the body size of intertidal ectotherms, with consequences extending from populations to ecosystem functions and services. The consistency of our results on body size across species with different life histories, feeding modes and microhabitats inhabiting a single coastal ecosystem, and for one species, across hemispheres, suggests that there is potential to generalize predictions of ecosystem responses to climate change with important implications for conservation. Finally, our results illustrate the potential for the use of appropriately designed and constrained space-for-time substitutions across an ecological community to increase our understanding of the effects of global climate change on broadly distributed populations and communities of coastal ecosystems, particularly those for which robust time series data are not available.

## Supporting information

S1 File Raw data and estimations of predicted changes in body sizes. (XLSX)

S1 Table Names, codes and location of the sandy beaches studied at Chile and USA (California). (XLSX)

S2 Table Results of regression analyses of SST and Chl a as a function of latitude in the coast of Chile and USA (California). (XLSX)

S3 Table Results of regression analyses of beach features as a function of latitude in Chile and USA (California). (XLSX)

S4 Table Predicted change in body size, fecundity and biomass of two intertidal species of Chile and USA (California) as a function of changes in surf zone SST. (XLSX)

## References

[1] Walther G-R, Post E, Convey P, Menzel A, Parmesan C, Beebee TJC, et al Ecological responses to recent climate change. Nature. Nature Publishing Group; 2002;416: 389–395. doi: 10.1038/416389a 11919621 10.1038/416389a

[2] Thomas CD, Cameron A, Green RE, Bakkenes M, Beaumont LJ, Collingham YC, et al Extinction risk from climate change. Nature. Nature Publishing Group; 2004;427: 145–148. doi: 10.1038/nature02121 14712274 10.1038/nature02121

[3] Parmesan C. Ecological and evolutionary responses to recent climate change. Annu Rev Ecol Evol Syst. JSTOR; 2006; 637–669.

[4] Walther G-R. Community and ecosystem responses to recent climate change. Philos Trans R Soc B Biol Sci. The Royal Society; 2010;365: 2019–2024. 10.1098/rstb.2010.0021 PMC2880129 20513710

[5] García Molinos J, Halpern BS, Schoeman DS, Brown CJ, Kiessling W, Moore PJ, et al Climate velocity and the future global redistribution of marine biodiversity. Nat Clim Chang. Nature Publishing Group; 2015;6: 83–88.

[6] Poloczanska ES, Brown CJ, Sydeman WJ, Kiessling W, Schoeman DS, Moore PJ, et al Global imprint of climate change on marine life. Nat Clim Chang. Nature Publishing Group; 2013;3: 919–925.

[7] Poloczanska ES, Burrows MT, Brown CJ, Garcia Molinos J, Halpern BS, Hoegh-Guldberg O, et al Responses of marine organisms to climate change across oceans. Front Mar Sci. Frontiers; 2016;3: 62.

[8] Loeb V, Siegel V, Holm-Hansen O, Hewitt R, Fraser W, Trivelplece W, et al Effects of sea-ice extent and krill or salp dominance on the Antarctic food web. Nature. Nature Publishing Group; 1997;387: 897–900.

[9] Hoegh-Guldberg O. Climate change, coral bleaching and the future of the world’s coral reefs. Mar Freshw Res. CSIRO; 1999;50: 839–866.

[10] Perry AL, Low PJ, Ellis JR, Reynolds JD. Climate change and distribution shifts in marine fishes. Science. American Association for the Advancement of Science; 2005;308: 1912–1915. doi: 10.1126/science.1111322 15890845 10.1126/science.1111322

[11] Jansen JM, Pronker AE, Bonga SW, Hummel H. Macoma balthica in Spain, a few decades back in climate history. J Exp Mar Bio Ecol. Elsevier; 2007;344: 161–169.

[12] Sorte FA La Frank III RT. Poleward shifts in winter ranges of North American birds. Ecology. Wiley Online Library; 2007;88: 1803–1812. 17645026 10.1890/06-1072.1

[13] Moritz C, Patton JL, Conroy CJ, Parra JL, White GC, Beissinger SR. Impact of a century of climate change on small-mammal communities in Yosemite National Park, USA. Science. American Association for the Advancement of Science; 2008;322: 261–264. doi: 10.1126/science.1163428 18845755 10.1126/science.1163428

[14] Wethey DS, Woodin SA. Ecological hindcasting of biogeographic responses to climate change in the European intertidal zone. Hydrobiologia. Springer; 2008;606: 139–151.

[15] Burrows MT, Schoeman DS, Buckley LB, Moore P, Poloczanska ES, Brander KM, et al The pace of shifting climate in marine and terrestrial ecosystems. Science. American Association for the Advancement of Science; 2011;334: 652–655. doi: 10.1126/science.1210288 22053045 10.1126/science.1210288

[16] Burrows MT, Schoeman DS, Richardson AJ, Molinos JG, Hoffmann A, Buckley LB, et al Geographical limits to species-range shifts are suggested by climate velocity. Nature. Nature Publishing Group; 2014;507: 492–495. doi: 10.1038/nature12976 24509712 10.1038/nature12976

[17] Lima FP, Wethey DS. Three decades of high-resolution coastal sea surface temperatures reveal more than warming. Nat Commun. Nature Publishing Group; 2012;3. 10.1038/ncomms1713 22426225

[18] Harley CDG, Randall Hughes A, Hultgren KM, Miner BG, Sorte CJB, Thornber CS, et al The impacts of climate change in coastal marine systems. Ecol Lett. Wiley Online Library; 2006;9: 228–241. doi: 10.1111/j.1461-0248.2005.00871.x 16958887 10.1111/j.1461-0248.2005.00871.x

[19] Schlacher TA, Dugan J, Schoeman DS, Lastra M, Jones A, Scapini F, et al Sandy beaches at the brink. Divers Distrib. Wiley Online Library; 2007;13: 556–560.

[20] Doney SC, Ruckelshaus M, Duffy JE, Barry JP, Chan F, English CA, et al Climate change impacts on marine ecosystems. Mar Sci. 2012;4. 10.1146/annurev-marine-041911-111611 22457967

[21] Schoeman DS, Schlacher TA, Defeo O. Climate‐change impacts on sandy‐beach biota: crossing a line in the sand. Glob Chang Biol. Wiley Online Library; 2014;20: 2383–2392. 25121188 10.1111/gcb.12505

[22] Gardner JL, Peters A, Kearney MR, Joseph L, Heinsohn R. Declining body size: a third universal response to warming? Trends Ecol Evol. Elsevier; 2011;26: 285–291. doi: 10.1016/j.tree.2011.03.005 21470708 10.1016/j.tree.2011.03.005

[23] Sheridan JA, Bickford D. Shrinking body size as an ecological response to climate change. Nat Clim Chang. Nature Publishing Group; 2011;1: 401–406.

[24] Millien V, Kathleen Lyons S, Olson L, Smith FA, Wilson AB, Yom‐Tov Y. Ecotypic variation in the context of global climate change: revisiting the rules. Ecol Lett. Wiley Online Library; 2006;9: 853–869. doi: 10.1111/j.1461-0248.2006.00928.x 16796576 10.1111/j.1461-0248.2006.00928.x

[25] Daufresne M, Lengfellner K, Sommer U. Global warming benefits the small in aquatic ecosystems. Proc Natl Acad Sci. National Acad Sciences; 2009;106: 12788–12793. doi: 10.1073/pnas.0902080106 19620720 10.1073/pnas.0902080106 PMC2722360

[26] Yvon-Durocher G, Montoya JM, Trimmer M, Woodward GUY. Warming alters the size spectrum and shifts the distribution of biomass in freshwater ecosystems. Glob Chang Biol. Wiley Online Library; 2011;17: 1681–1694.

[27] Cheung WWL, Sarmiento JL, Dunne J, Frölicher TL, Lam VWY, Palomares MLD, et al Shrinking of fishes exacerbates impacts of global ocean changes on marine ecosystems. Nat Clim Chang. Nature Publishing Group; 2013;3: 254–258.

[28] Garzke J, Ismar SMH, Sommer U. Climate change affects low trophic level marine consumers: warming decreases copepod size and abundance. Oecologia. Springer; 2015;177: 849–860. doi: 10.1007/s00442-014-3130-4 25413864 10.1007/s00442-014-3130-4

[29] Forster J, Hirst AG, Atkinson D. Warming-induced reductions in body size are greater in aquatic than terrestrial species. Proc Natl Acad Sci. National Acad Sciences; 2012;109: 19310–19314. doi: 10.1073/pnas.1210460109 23129645 10.1073/pnas.1210460109 PMC3511100

[30] Berke SK, Jablonski D, Krug AZ, Roy K, Tomasovych A. Beyond Bergmann's rule: size–latitude relationships in marine Bivalvia world-wide. Global Ecol. Biogeogr; 2013; 22, 173–183

[31] Roy K, Collins AG, Becker BJ, Begovic E, Engle JM. Anthropogenic impacts and historical decline in body size of rocky intertidal gastropods in southern California. Ecol Lett. Wiley Online Library; 2003;6: 205–211.

[32] Meiri S, Guy D, Dayan T, Simberloff D. Global change and carnivore body size: data are stasis. Glob Ecol Biogeogr. Wiley Online Library; 2009;18: 240–247.

[33] Lester RE, Close PG, Barton JL, Pope AJ, Brown SC. Predicting the likely response of data-poor ecosystems to climate change using space-for-time substitution across domains; 2014; Glob Change Biol 20: 3471−3481 10.1111/gcb.12634 24832685

[34] Weymouth FW, McMillin HC, Rich WH. Latitude and relative growth in the razor clam, Siliqua patula. J Exp Biol. The Company of Biologists Ltd; 1931;8: 228–249.

[35] Jones MB, Simons MJ. Latitudinal variation in reproductive characteristics of a mud crab, Helice crassa (Grapsidae). Bull Mar Sci. University of Miami-Rosenstiel School of Marine and Atmospheric Science; 1983;33: 656–670.

[36] Dugan JE, Wenner AM, Hubbard DM. Geographic variation in the reproductive biology of the sand crab Emerita analoga (Stimpson) on the California coast. J Exp Mar Bio Ecol. Elsevier; 1991;150: 63–81.

[37] Dugan JE, Hubbard DM, Wenner AM. Geographic variation in life history of the sand crab, Emerita analoga (Stimpson) on the California coast: relationships to environmental variables. J Exp Mar Bio Ecol. Elsevier; 1994;181: 255–278.

[38] Pickett S. Space-for-time substitution as an alternative to long-term studies In: Likens GE (ed) Long-term studies in ecology: approaches and alternatives; 1989; Springer, New York, NY, p 110−135

[39] Celantano E, Defeo O. Effects of climate on the mole crab *Emerita brasiliensis* on a dissipative beach in Uruguay. Mar Ecol Prog Ser; 2016 552: 211–222

[40] Bascom W. Waves and beaches; the dynamics of the ocean surface. Anchor Books; 1964.

[41] Dugan JE, Defeo O, Jaramillo E, Jones AR, Lastra M, Nel R, Peterson CH, Scapini F, Schlacher T, Schoeman DS. 2010 Give beach ecosystems their day in the sun. Science, 329: 1146 10.1126/science.329.5996.1146-a 20813935

[42] Dugan JE, Hubbard DM, Quigley BJ. Beyond beach width: Steps toward identifying and integrating ecological envelopes with geomorphic features and datums for sandy beach ecosystems. Geomorphology. Elsevier; 2013;199: 95–105.

[43] Helmuth B, Harley CDG, Halpin PM, O’Donnell M, Hofmann GE, Blanchette CA. Climate change and latitudinal patterns of intertidal thermal stress. Science. American Association for the Advancement of Science; 2002;298: 1015–1017. doi: 10.1126/science.1076814 12411702 10.1126/science.1076814

[44] Sydeman WJ, García-Reyes M, Schoeman DS, Rykaczewski RR, Thompson SA, Black BA, et al Climate change and wind intensification in coastal upwelling ecosystems. Science. American Association for the Advancement of Science; 2014;345: 77–80. doi: 10.1126/science.1251635 24994651 10.1126/science.1251635

[45] Jaramillo E, Contreras H, Duarte C, Quijon P. Relationships Between Community Structure of the Intertidal Macroinfauna and Sandy Beach Characteristics Along the Chilean Coast. Mar Ecol. Wiley Online Library; 2001;22: 323–342.

[46] Dugan JE, Hubbard DM, McCrary MD, Pierson MO. The response of macrofauna communities and shorebirds to macrophyte wrack subsidies on exposed sandy beaches of southern California. Estuar Coast Shelf Sci. Elsevier; 2003;58: 25–40.

[47] Marincovich L. Intertidal mollusks of Iquique, Chile. Natural History Museum, Los Angeles County; 1973.

[48] Valentine JW. Numerical analysis of marine molluscan ranges on the extratropical northeastern Pacific shelf. Limnol Oceanogr. Wiley Online Library; 1966;11: 198–211.

[49] Duarte C, Navarro JM, Acuña K, Gómez I. Feeding preferences of the sandhopper Orchestoidea tuberculata: the importance of algal traits. Hydrobiologia. Springer; 2010;651: 291–303.

[50] Jaramillo E, Mclachlan A. Community and Population Responses of the Macroinfauna to Physical Factors over a Range of Exposed Sandy in South-central Chile. Estuar Coast Shelf Sci. 1993;37: 615–624.

[51] Contreras H, Jaramillo E. Geographical variation in natural history of the sandy beach isopod Excirolana hirsuticauda Menzies (Cirolanidae) on the Chilean coast. Estuar Coast Shelf Sci. 2003;58: 117–126.

[52] Johnson MW, Lewis WM. Pelagic Larval Stages of the Sand Crabs Emerita analoga (Stimpson), Blepharipoda occidentalis Randall, and Lepidopa myops Stimpson. Biol Bull. 1942;83: 67.

[53] Efford IE. Aggregation in the sand crab, Emerita analoga (Stimpson). J Anim Ecol. JSTOR; 1965; 63–75.

[54] Efford IE. Recruitment to sedentary marine populations as exemplified by the sand crab, Emerita analoga (Decapoda, Hippidae). Crustaceana. Brill; 1970;18: 293–308.

[55] Jaramillo E. Sandy Beach Macroinfauna from the chilean coast: Zonation patterns and zoogeography. Vie Milieu. 1987;37: 165–174.

[56] Glynn PW, Dexter DM, Bowman TE. Excirolana braziliensis, a Pan-American isopod: taxonomic status, zonation and distribution. J Zool. 1975;175: 509–521.

[57] Weinberg JR, Starczak VR. Morphological divergence of Eastern Pacific and Caribbean isopods: effects of a land barrier and the Panama Canal. Mar Biol. Springer; 1989;103: 143–152.

[58] Varela AI, Haye PA. The marine brooder Excirolana braziliensis (Crustacea: Isopoda) is also a complex of cryptic species on the coast of Chile. Rev Chil Hist Nat. Sociedad de Biología de Chile; 2012;85: 495–502.

[59] Efford IE. Distribution of the sand crabs in the genus Emerita (Decapoda, Hippidae). Crustaceana. JSTOR; 1976; 169–183.

[60] Wenner AM, Hubbard DM, Dugan J, Shoffner J, Jellison K. Egg production by sand crabs (Emerita analoga) as a function of size and year class (Decapoda, Hippidae). Biol Bull. MBL; 1987;172: 225–235.

[61] Strickland JD, Parsons TR. A practical handbook of seawater analysis, 2^nd^ edition Bulletin Fisheries Research Board of Canada; 1972; 167:310 pp

[62] Seward-Thompson BL, Hails JR. An appraisal of the computation of statistical parameters in grain size analysis. Sedimentology. 1973;20: 161–169.

[63] Folk R. Petrology of sedimentary rocks. Austin, Texas: Hemphill Publishing Company; 1980.

[64] Gibbs RJ, Matthews MD, Link DA. The relationship between sphere size and settling velocity. J Sediment Res. SEPM; 1971;41: 7–18.

[65] Dugan J, Hubbard DM. Local variation in populations of the sand crab Emerita analoga on sandy beaches in southern California. Rev Chil Hist Nat. 1996;69: 579–588.

[66] Short AD, Wright LD. Physical variability of sandy beaches Sandy beaches as ecosystems. Springer; 1983 pp. 133–144.

[67] R-Core-Team. R: A language and environment for statistical computing. Vienna, Austria; 2013.

[68] Contreras H, Defeo O, Jaramillo E. Life History of Emerita analoga (Stimpson) (Anomura, Hippidae) in a Sandy Beach of South Central Chile. Estuar Coast Shelf Sci. 1999;48: 101–112.

[69] Reynolds RW, Rayner NA, Smith TM, Stokes DC, Wang W. An Improved In Situ and Satellite SST Analysis for Climate. 2002.

[70] Bakun A. Global climate change and intensification of coastal ocean upwelling. Science. 1990;247: 198–201. doi: 10.1126/science.247.4939.198 17813287 10.1126/science.247.4939.198

[71] Tomašových A, Jablonski D, Berke SK, Krug AZ, Valentine JW. Nonlinear thermal gradients shape broad-scale patterns in geographic range size and can reverse Rapoport’s rule. Global Ecol. Biogeogr; 2015; 24, 157–167

[72] Dugan JE. Geographic and temporal variation in the life history, growth and reproductive biology of the sand crab, Emerita analoga (Stimpson). University of California, Santa Barbara 1990.

[73] Gaston KJ, Chown SL, Evans KL. Ecogeographical rules: elements of a synthesis. J Biogeogr. 2008;35: 483–500.

[74] Mayr E, Mayr E, Mayr E, Mayr E. Animal species and evolution. Belknap Press of Harvard University Press Cambridge, Massachusetts; 1963.

[75] Blackburn TM, Gaston KJ, Loder N. Geographic gradients in body size: a clarification of Bergmann’s rule. Divers an Distrib. 1999;5: 165–174.

[76] Atkinson D. Temperature and organism size: a biological law for ectotherms? Adv Ecol Res. Academic Press; 1994;25: 1.

[77] Ray C. The application of Bergmann’s and Allen’s rules to the poikilotherms. J Morphol. Wiley Online Library; 1960;106: 85–108. doi: 10.1002/jmor.1051060104 14436612 10.1002/jmor.1051060104

[78] James FC. Geographic size variation in birds and its relationship to climate. Ecology. Wiley Online Library; 1970;51: 365–390.

[79] Meiri S, Dayan T. On the validity of Bergmann’s rule. J Biogeogr. Wiley Online Library; 2003;30: 331–351.

[80] Ashton KG. Sensitivity of intraspecific latitudinal clines of body size for tetrapods to sampling, latitude and body size. Integr Comp Biol. Soc Integ Comp Biol; 2004;44: 403–412. doi: 10.1093/icb/44.6.403 21676726 10.1093/icb/44.6.403

[81] Chown SL, Gaston KJ. Body size variation in insects: a macroecological perspective. Biol Rev. Wiley Online Library; 2010;85: 139–169. doi: 10.1111/j.1469-185X.2009.00097.x 20015316 10.1111/j.1469-185X.2009.00097.x

[82] Frank PW. Latitudinal variation in the life history features of the black turban snail Tegula funebralis (Prosobranchia: Trochidae). Mar Biol. Springer; 1975;31: 181–192.

[83] Annala JH, McKoy JL, Booth JD, Pike RB. Size at the onset of sexual maturity in female Jasus edwardsii (Decapoda: Palinuridae) in New Zealand. New Zeal J Mar Freshw Res. 1980;14: 217–227.

[84] Kelley AL, de Rivera CE, Grosholz ED, Ruiz GM, Yamada SB, Gillespie G. Thermogeographic variation in body size of Carcinus maenas, the European green crab. Mar Biol; 2015; 162:1625–1635

[85] Osorio C, Bahamonde N, Lopez M. El Limanche (Emerita analoga, Stimpson) en Chile. Bol del Mus Nac Hist Nat Santiago, Chile. 1971;29: 61–116.

[86] Cardoso RS, Defeo O. Biogeographic patterns in life history traits of the Pan-American sandy beach isopod Excirolana braziliensis. Estuar Coast Shelf Sci. Elsevier; 2004;61: 559–568.

[87] Manyak-Davis A, Bell TM, Sotka EE. The relative importance of predation risk and water temperature in maintaining Bergmann’s Rule in a marine ectotherm. The American Naturalist; 2013; 182: 347–358. doi: 10.1086/671170 23933725 10.1086/671170

[88] Chapelle G, Peck LS. Polar gigantism dictated by oxygen availability. Nature. Nature Publishing Group; 1999;399: 114–115.

[89] Weber MJ, Brown ML, Wahl DH, Shoup DE. Metabolic theory explains latitudinal variation in common carp populations and predicts responses to climate change. Ecosphere. Wiley Online Library; 2015;6: 1–16.

[90] Hartnoll RG. Growth, sexual maturity and reproductive output. Crustac issues. A. Balkema Rotterdam; 1985;3: 101–128.

[91] March BGE de. The effects of constant and variable temperatures on the size, growth, and reproduction of the freshwater amphipod Hyalella azteca (Saussure). Can J Zool. NRC Research Press; 1978;56: 1801–1806.

[92] Thomas AC, Carr M, Strub PT. Chlorophyll variability in eastern boundary currents. Geophys Res Lett. Wiley Online Library; 2001;28: 3421–3424.

[93] Pinsky ML, Worm B, Fogarty MJ, Sarmiento JL, Levin SA. Marine taxa track local climate velocities. Science. American Association for the Advancement of Science; 2013;341: 1239–1242. doi: 10.1126/science.1239352 24031017 10.1126/science.1239352

[94] Bates AE, Pecl GT, Frusher S, Hobday AJ, Wernberg T, Smale DA, et al Defining and observing stages of climate-mediated range shifts in marine systems. Glob Environ Chang. Elsevier; 2014;26: 27–38.

[95] Sunday JM, Pecl GT, Frusher S, Hobday AJ, Hill N, Holbrook NJ, et al Species traits and climate velocity explain geographic range shifts in an ocean warming hotspot. Ecol Lett. Wiley Online Library; 2015;18: 944–953. doi: 10.1111/ele.12474 26189556 10.1111/ele.12474

[96] Gillson L, Dawson TP, Jack S, McGeoch MA. Accommodating climate change contingencies in conservation strategy. Trends Ecol Evol. Elsevier; 2013;28: 135–142. doi: 10.1016/j.tree.2012.10.008 23146578 10.1016/j.tree.2012.10.008

[97] Parmesan C, Burrows MT, Duarte CM, Poloczanska ES, Richardson AJ, Schoeman DS, et al Beyond climate change attribution in conservation and ecological research. Ecol Lett. Wiley Online Library; 2013;16: 58–71. doi: 10.1111/ele.12098 23679010 10.1111/ele.12098

[98] Lastra M, Page HM, Dugan JE, Hubbard DM, Rodil IF. Processing of allochthonous macrophyte subsidies by sandy beach consumers: estimates of feeding rates and impacts on food resources. Mar Biol. Springer; 2008;154: 163–174.

